# RNA and Sugars, Unique Properties of Bacteriophages Infecting Multidrug Resistant *Acinetobacter radioresistens* Strain LH6

**DOI:** 10.3390/v13081652

**Published:** 2021-08-20

**Authors:** Clay S. Crippen, Bibi Zhou, Silke Andresen, Robert T. Patry, Artur Muszyński, Craig T. Parker, Kerry K. Cooper, Christine M. Szymanski

**Affiliations:** 1 Department of Microbiology, University of Georgia, Athens, GA 30602, USA; clay.crippen25@uga.edu (C.S.C.); Bibi.Zhou@uga.edu (B.Z.); Silke.Andresen1@uga.edu (S.A.); rpatry41218@gmail.com (R.T.P.); 2 Complex Carbohydrate Research Center, University of Georgia, Athens, GA 30602, USA; muszynski@ccrc.uga.edu; 3 Produce Safety and Microbiology Research Unit, Western Regional Research Center, Agricultural Research Service, U.S. Department of Agriculture, Albany, CA 94710, USA; Craig.Parker@usda.gov; 4 School of Animal and Comparative Biomedical Sciences, University of Arizona, Tucson, AZ 85721, USA; kcooper@arizona.edu

**Keywords:** *Acinetobacter*, bacteriophages, segmented RNA viruses, pilin, capsular polysaccharides, lipooligosaccharides

## Abstract

Bacteriophages (phages) are predicted to be the most ubiquitous biological entity on earth, and yet, there are still vast knowledge gaps in our understanding of phage diversity and phage–host interactions. Approximately one hundred *Acinetobacter*-infecting DNA viruses have been identified, and in this report, we describe eight more. We isolated two typical dsDNA lytic podoviruses (CAP1–2), five unique dsRNA lytic cystoviruses (CAP3–7), and one dsDNA lysogenic siphovirus (SLAP1), all capable of infecting the multidrug resistant isolate *Acinetobacter radioresistens* LH6. Using transmission electron microscopy, bacterial mutagenesis, phage infectivity assays, carbohydrate staining, mass-spectrometry, genomic sequencing, and comparative studies, we further characterized these phages. Mutation of the LH6 initiating glycosyltransferase homolog, PglC, necessary for both O-linked glycoprotein and capsular polysaccharide (CPS) biosynthesis, prevented infection by the lytic podovirus CAP1, while mutation of the pilin protein, PilA, prevented infection by CAP3, representing the lytic cystoviruses. Genome sequencing of the three dsRNA segments of the isolated cystoviruses revealed low levels of homology, but conserved synteny with the only other reported cystoviruses that infect *Pseudomonas* species. In *Pseudomonas*, the cystoviruses are known to be enveloped phages surrounding their capsids with the inner membrane from the infected host. To characterize any membrane-associated glycoconjugates in the CAP3 cystovirus, carbohydrate staining was used to identify a low molecular weight lipid-linked glycoconjugate subsequently identified by mutagenesis and mass-spectrometry as bacterial lipooligosaccharide. Together, this study demonstrates the isolation of new *Acinetobacter*-infecting phages and the determination of their cell receptors. Further, we describe the genomes of a new genus of *Cystoviruses* and perform an initial characterization of membrane-associated glycoconjugates.

## 1. Introduction

Bacteriophages (phages) are viruses that replicate in bacterial hosts and have been gaining attention for their potential use in biocontrol applications [1,2]. Phages are estimated to infect every bacterial genus, outnumbering bacteria by approximately tenfold [3]. Despite the overwhelming number of phages present in the biosphere, very few studies provide detailed descriptions of phage dynamics with their hosts. 

The genus *Acinetobacter* has approximately 100 identified and 37 sequenced phages [4]. The majority of these phages belong to the dsDNA *Caudovirales* and infect the emerging pathogen *Acinetobacter baumannii*. This species is at the top of the list provided by the World Health Organization and the Centers for Disease Control and Prevention describing pathogens for which new antimicrobials are most desperately needed [5]. Thus, the majority of phage screens have been directed toward this pathogen, and the host range for these *Acinetobacter*-infecting phages will be limited to *A. baumannii*. Recently, we isolated the *Acinetobacter radioresistens* strain LH6 from chicken feces on a free-range farm [6]. We found that this multidrug resistant reservoir strain encodes genes associated with resistance to toxic metals and quaternary ammonium compounds [7]. We also confirmed the presence of the infamous *bla* oxacillinase gene, which has been directly linked to extreme drug resistance and is believed to originate from this species, although LH6 lacks the accessory insertional element necessary to induce expression of the gene [7]. In addition, LH6 is capable of tolerating desiccation for significantly longer periods of time compared to *A. baumannii* and *Escherichia coli* [8], with reports describing desiccation revival after >160 days and tolerance to radiation for this species [9].

**Figure 1:**
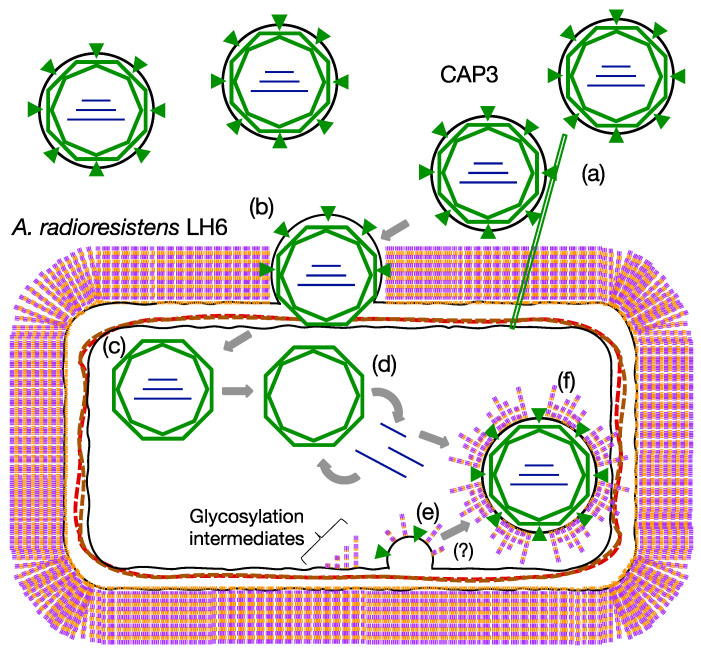
Proposed schematic life cycle of *A. radioresistens* infecting cystovirus CAP3. (**a**) CAP3 binds to the protruding pilus. (**b**) CAP3 fuses with the host outer membrane, degrading host peptidoglycan with its muramidase. (**c**) CAP3 capsid enters the cytoplasm. (**d**) dsRNA segments are polymerized out of the capsids as +ssRNA (prior to entry into a new capsid for dsRNA synthesis) and viral replication takes place. (**e**) The cell inner membrane, the site of most bacterial glycoconjugate biosynthetic assemblies, is used to envelop virion particles. (**f**) Mature virion particles are constructed, potentially with intermediates of host glycosylation embedded in the membrane, ready to lyse the cell and be released.

One contributing factor to the exceptional environmental stress tolerance of LH6, and *Acinetobacter* strains more generally, are their diverse glycan structures [10,11]. Acinetobacters possess pathways for the biosynthesis of capsular polysaccharides (CPS), trehalose [12] lipooligosaccharides (LOS), poly-N-acetylglucosamine (PNAG), and O-linked glycoproteins (modified with O-glycans) [10], all of which can play significant roles in *Acinetobacter* persistence and virulence [10]. In *A. baumannii,* CPS and O-glycans use a common pathway to assemble oligosaccharides onto the lipid carrier undecaprenylphosphate (UndP) in the inner leaflet of the inner membrane before being flipped into the periplasmic space and transferred to proteins (including pilin) or polymerized into CPS and exported to the outer membrane [13,14]. Similarly, the LOS is produced via the Raetz pathway, wherein Kdo_2_-lipid A is built in the inner leaflet of the inner membrane and the oligosaccharide core is assembled sequentially onto the first Kdo sugar moiety, yielding LOS. The LOS is then flipped into the periplasmic space and transferred to the outer membrane via the Lpt pathway [15], readily recognizable from the genomic sequence of LH6 [6]. Another sugar structure that is commonly produced by acinetobacters is the PNAG polymer. PNAG is formed by polymerizing N-acetylglucosamine and releasing the polysaccharide into the extracellular matrix during biofilm assembly and to aid in adherence to surfaces [16]. Together, all of these sugar structures contribute to the tenacity and pathogenesis of *Acinetobacter* species.Along with the isolation of LH6, we conducted phage screens using fecal samples collected from poultry species housed on the same farm. We isolated several phages classifying into three different families, including an induced lysogenic syphovirus and two lytic podoviruses, all infecting strain LH6. Amongst these phages, we also isolated the first reported group of segmented dsRNA phages in a species outside of the genus *Pseudomonas*. Before this report, there were seven sequenced pseudomonad dsRNA phages in the International Committee on Taxonomy of Viruses (ICTV) database constituting the family *Cystoviridae* [17]. Cystoviruses are viruses with a life cycle unique to phages, which has been studied extensively in the type phage Phi6 [18]. In general, they are composed of a tripartite segmented dsRNA genome within two capsids enveloped in the inner membrane of the host in which they last replicated. After infecting through the pilus or rough lipopolysaccharide (LPS, or what we refer to as LOS) of the host, the dsRNA segments are polymerized while shielded by the incomplete capsid as polycistronic mRNA, limiting exposure of dsRNA, which is foreign to host nucleases and could be destroyed [18]. After viral replication, the RNA segments are packed into assembled capsids where the genome is replicated by integral RNA polymerases. The phage capsid is then enveloped in the host inner membrane before degrading the peptidoglycan, resulting in lysis of the outer membrane and exiting out of the host cell (Figure 1) [17].

In this study, we sequenced all five of the isolated cystoviruses and selected one, CAP3, for further characterization. We were particularly interested in whether the host-derived envelope of CAP3 captured any glycan intermediates known to be actively assembled at the inner membrane into the diverse array of glycan structures that are subsequently exported in the host. To date, no host-associated structural glycoconjugates have been described for these or other small bacteriophages. Knowing that the membranes of *Acinetobacter* are rich in lipid-linked glycans we hypothesized that the phage would coat itself in host membranes containing these glycoconjugates. We also mutated select LH6 glycan biosynthesis gene homologs to assess their impact on phage infection and potential envelope modification. Together, these results describe three diverse and one novel *Acinetobacter*-infecting phage genera with several unexpected features requiring further study.

## 2. Materials and Methods

### 2.1. Bacterial Growth Conditions

*A. radioresistens* strain LH6 was grown aerobically using Luria–Bertani (LB) medium (Becton, Dickinson and Company, Franklin Lakes, NJ, USA) at 30 °C and under agitation at 200 rpm for liquid culture. In phage propagation conditions, brain heart infusion (BHI) medium (Hardy Diagnostics, Santa Maria, CA, USA) was used to increase yields.

### 2.2. Lytic Bacteriophage Propagation and Isolation

Bacteriophage isolation was performed as described [19]. Propagating strain LH6 was grown overnight at 30 °C with shaking at 200 rpm, the culture was adjusted to OD_600_ = 0.3, and infected with bacteriophages at a multiplicity of infection (MOI) of 0.001. The infected culture was incubated at 30 °C with shaking at 200 rpm overnight. Afterward, the culture was centrifuged at 4255× *g* for 15 min, the resulting supernatant was filtered through a 0.22 μm filter and the phage-containing filtrate was collected.

### 2.3. Lysogenic SLAP1 Bacteriophage Induction and Isolation

For SLAP1 prophage induction, propagating strain LH6 was grown in BHI broth overnight at 30 °C, shaking at 200 rpm. The culture was adjusted to OD_600_ of 0.05 and was grown overnight in the presence of 1 µg/mL mitomycin C (Gold-Biotechnology, St. Louis, MO, USA). After centrifugation at 7020× *g* for 10 min, the phage-containing supernatant was filtered (0.2 µm), and the phage-containing filtrate was collected. The filtered supernatant was then ultra-centrifuged at 141,000× *g* for 1.5 h at 4 °C. The resulting phage pellet was resuspended in SM buffer (100 mM NaCl, 8 mM MgSO_4_•7H_2_O, 50 mM Tris-HCl pH 7.5, 0.002% (*w*/*v*) gelatin). For high-resolution transmission electron microscopy (TEM) imaging, the concentrated prophages were treated with chloroform. Briefly, 0.1 volume of chloroform was added to the phage solution, vortexed occasionally and incubated for 10 min at RT. The suspension was centrifuged at 4000× *g* for 5 min, the top layer containing the phages was transferred to a new tube and imaged as described below. For the clearance assay, this was done similarly to the spot assay described previously [7]. Briefly, the host strain LH6 was grown overnight and adjusted to OD_600_ = 0.3. Then, 500 µL bacteria, 5 mL of molten BHI 0.6% agar, and in the case of SLAP1, 1 µg/mL mitomycin C, were mixed and poured onto a BHI agar plate. After the agar solidified, 10 µL of the phage suspension was spotted onto the medium and the plate was incubated overnight. 

### 2.4. Bacteriophage Transmission Electron Microscopy 

Following propagation, bacteriophages CAP1 and CAP3 were incubated for 5 min at room temperature with host strain LH6 (OD_600_ = 0.5) at MOI of ~100. Similarly, 50 µL of concentrated SLAP1 was incubated for 2 min with 250 µL LH6 cells at OD_600_ = 0.5. Then 5% paraformaldehyde (Electron Microscopy Sciences, Hatfield, PA, USA) was added to fix the samples and halt the infection. The samples were spotted onto parafilm and formvar-coated copper grids (Electron Microscopy Sciences) were placed on top of each drop and left to incubate for 1 h at room temperature, followed by three 3 min washes in each 1x PBS and ddH_2_O. The grids were negatively stained with 0.5% phosphotungstic acid (Electron Microscopy Sciences) for 10–30 s, wicked onto a Kimwipe and imaged with the JEOL JEM1011 TEM (JEOL Inc., Akishima, Tokyo, Japan).

### 2.5. Genome Characterization

Phage genomes were isolated by the phenol/chloroform method described [20]. To determine the nucleic acid composition, 1 μg of each genome was incubated with DNase I or RNase A (ThermoFisher Scientific, Waltham, MA, USA) according to the manufacturer’s instructions, separated on a 1% agarose gel and imaged. To determine ds or ssRNA in CAP3, RNase I_f_ (New England BioLabs, Ipswich, MA, USA) was added per manufacturer’s instructions to the ssRNA ladder (New England BioLabs), dsRNA ladder (New England BioLabs) and CAP3 genomic RNA. The reaction was stopped with 0.1% sodium dodecyl sulfate (final volume) and separated on a 2% agarose gel and imaged.

### 2.6. CAP3–CAP7 Whole Genome Sequencing

Phage genomes were provided to GENEWIZ for cDNA library construction and Illumina sequencing using 150 bp reads with >100x coverage. Illumina reads were quality filtered (≥Q30) using Geneious Prime (v2020.1), and then high-quality filtered reads were mapped to the *A. radioresistens* LH6 genome to eliminate any reads from rRNA loci or other LH6 gene expression. Next, unmapped reads were assembled into contigs using Newbler assembler (v2.6), and the resulting contigs were cleaned and the finalized chromosome assembly for CAP3–7 was done using Geneious Prime.

The evolutionary history was inferred by using the Maximum Likelihood method based on the Kimura 2-parameter model [21]. The trees with the highest log likelihood (segment 1: −10401.29, segment 2: −4365.84, segment 3: −6612.83) are shown. The percentage of trees in which the associated taxa clustered together are shown next to the branches. Initial tree(s) for the heuristic search were obtained automatically by applying Neighbor-Join and BioNJ algorithms to a matrix of pairwise distances estimated using the Maximum Composite Likelihood (MCL) approach, and then selecting the topology with superior log likelihood value. The trees are drawn to scale, with branch lengths measured in the number of substitutions per site. The analysis involved 5 nucleotide sequences. There were a total of 6614 positions (base pairs) for segment 1, 2738 positions for segment 2, and 3666 positions for segment 3 in the final dataset. Evolutionary analyses were conducted in MEGA7 [22].

### 2.7. Identification of Induced Prophage SLAP1

For the identification of the induced prophage SLAP1, primers were designed that target the capsid genes of prophage 1 and prophage 2 [6]. Prophage 1 was detected with the primers pP1capsid-F and pP1capsid-R, prophage 2 was detected with the primers pP2capsid-F and pP2capsid-R (primers listed in Table S1). The PCR was performed with OneTaq polymerase (New England BioLabs) following the manufacturer’s protocol with the annealing step at 52 °C for 1 min and the elongation at 68 °C for 45 s. As template, 130 ng SLAP1 and LH6 genome were used, isolated via phenol/chloroform extraction and E.Z.N.A Bacterial DNA kit (Omega Bio-tek, Inc., Norcross, GA, USA), respectively. The PCR fragments were cleaned up via the DNA Clean & Concentrator-25 Kit (Zymo Research Inc., Irvine, CA, USA) and run on a 1% agarose gel for visualization.

### 2.8. Mutagenesis of LH6

The ∆*pglC* (DOM24_00295) knockout construct was made in pGEM-t-easy (Promega, Madison, WI, USA), with ~1000 bp homology upstream and downstream of *pglC* flanking either side of the kanamycin cassette from the plasmid pBAV1K-T5-*gfp* [23]. The primers (Table S1) used to generate the flanking regions were: ApaI-KO *pglC* upstream-F, SphI-KO *pglC* upstream-R, SpeI-RBS-KO *pglC* downstream-F, and PstI-KO *pglC* downstream-R. The ∆*pilA* (DOM24_13175) knockout construct was made in the same plasmid vector as for *pglC*. The ~900 bp upstream *pilA* mutant construct was fused with the kanamycin cassette promoter to ensure the function of the upstream gene, the primers used for this fragment were: ApaI-KO *pilA* upstream-F, NcoI-KO *pilA* upstream-R, KO *pilA* upstream with kan pro-F, and KO *pilA* upstream with kan pro-R. The primers for the ~1000 bp homology downstream were: SalI-KO *pilA* downstream-F, and SacI-KO *pilA* downstream-R. The ∆*lpsC* (DOM24_02210) and *clsB* (DOM24_06510) knockout constructs were made in the same plasmid vector. In the *lpsC* knockout construct, the ~1000 bp homology upstream and downstream were made by primers: ApaI-KO *lpsC* upstream-F, SphI-KO *lpsC* upstream-R, SpeI-RBS-KO *lpsC* downstream-F, and SalI-KO *lpsC* downstream-R. In the ∆*clsB* knockout construct, the ~1000 bp homology upstream and downstream were made by primers: SphI-KO *clsB* upstream-F, NcoI-KO *clsB* upstream-R, SpeI-RBS-KO *clsB* downstream-F, and SalI-KO *clsB* downstream-R. After construction of the knockout constructs, LH6 cells were mutated with an adaptation of the previously described recombineering method in *A. baumannii* [24]. Briefly, the plasmid pAT4 was inserted into LH6 and induced for four hours with 2 mM IPTG to express RecAB. The cells were then washed 3x with 10% glycerol and electroporated with a BioRad GenePulser on the Ec2 setting. The electroporated cells were then grown in 4 mL of liquid culture with 2 mM IPTG for 2 h before collecting the cells and plating on LB+ kanamycin (50 µg/mL) to select for mutants. The isolated mutants were then confirmed by PCR (Figure S1) and Sanger sequencing.

### 2.9. Purification of CAP Phages

Phages were propagated as described above on LH6 WT, ∆*pglC*, ∆*lpsC* and ∆*clsB* and the phage filtrate was collected. The phage was then purified with poly(ethylene glycol) 8000 (PEG 8000) precipitation and cesium chloride (CsCl) step-gradient protocols, adapted for use with these phages. The phage filtrate was incubated with 25 U/mL Turbonuclease (Sigma Aldrich, St. Louis, MO, USA) at room temperature for 30 min. Sodium chloride was added to a final concentration of 1 M, and the phage suspension was cooled in ice water for 1 h. Then, PEG 8000 was added to a final concentration of 10% and was dissolved with slow stirring. The phage suspension was transferred to centrifuge bottles and incubated at 4 °C overnight to allow phages to precipitate. After precipitation, phages were pelleted by centrifugation at 11,300× *g* for 25 min. The supernatant was removed and 5 mL sterile SM buffer (without gelatin) was added to saturate the pellets for 1 h at room temperature. The pellets were then resuspended and transferred to a sterile 15 mL tube for storage before purification in the CsCl step-gradient. 

The CsCl step-gradient was prepared by making CsCl solutions in sterile SM (without gelatin) at the following concentrations: 1.3 p = 0.404 g/mL; 1.5 p = 0.675 g/mL; 1.7 p = 0.943 g/mL. The layers were created in a Beckman SW-28 ultracentifuge polycarbonate insert tube by adding the least dense layer first and injecting denser layers on the bottom with a long needle attached to a syringe. The PEG-purified phages were added on top of the CsCl gradient and ultracentrifuged for 2.5 h at 25,000 rpm (~83,000× g) at 4 °C. After the phages were separated from impurities (appearing in the gradient as a blue-white band), the sidewall of the tube was punctured by an 18-gauge needle and the band was extracted. The phage solutions were then dialyzed using 3500 MWCO dialysis tubing in SM (without gelatin) overnight at 4 °C. The dialyzed phages were transferred to fresh SM for two additional hours before being removed from the dialysis tubing and filtered (0.2 µm), and were then stored at 4 °C. 

### 2.10. SDS-PAGE Analysis of Phage Glycans

Samples were standardized by protein content using a Pierce™ BCA kit (ThermoFisher) for CsCl-purified phages and by OD_600_ for bacterial cells, mixed with 5× SDS-loading dye (0.25% (*w*/*v*) bromophenol blue, 0.5 M DTT, 50% glycerol, 10% (*w*/*v*) SDS, 0.25 M Tris-HCl pH 6.8, 5% 2-mercaptoethanol) to a final concentration of 1×, heated at 95 °C for 15 min, and then loaded onto the 12.5% or 15% SDS-PAGE gels. Where indicated, proteinase K was added to a final concentration of 1 mg/mL and incubated with the samples for 18 h at 37 °C, followed by heating at 95 °C for 15 min and cooling to room temperature before loading onto the gels. Where indicated, glacial acetic acid was added to a final concentration of 1% and heated to 95 °C for 30 min. Proteins were separated by first running at 100 V for 10 min and then 170 V for 80 min. Samples were stained with the Pro-Q™ Emerald 300 (ThermoFisher) glycan staining kit according to manufacturer’s instructions. The same samples were also prepared without proteinase K treatment, separated on SDS-PAGE gels and stained with Coomassie stain.

### 2.11. Glycosyl and Fatty Acid Composition Analysis of CAP3 and LH6 by Gas Chromatography-Mass Spectrometry (GC-MS) 

CsCl-purified CAP3 and LH6 WT cells were prepared for glycosyl and fatty acid compositional analysis by first proteinase K treating (0.5 mg/mL) for 24 h at 37 °C, followed by 1 h at 60 °C. The proteinase K treated lysates were then dialyzed with a 1000 MWCO dialysis tubing into mqH_2_O before being lyophilized. Ten milligrams of cell or phage material was then dissolved into mqH_2_O and an equal volume of chloroform was added. The solution was vortexed for 30 s and phases were separated by centrifugation for 5 min at 4000× *g*. The aqueous phase was collected and an equal volume of fresh water was added; the process was repeated until three aqueous phases were collected. Then, the organic phase was collected in a similar manner for a total of three fractions. The interphase was collected last by dissolving in a small volume of water and chloroform. The water, chloroform and interphase samples were evaporated to dryness and the compositional analysis was performed by preparing trimethylsilyl (TMS) methyl glycoside derivatives after 18 h of methanolysis with 1 M HCl-MeOH at 80 °C. In addition to glycosyl composition, the methanolysis also allowed the conversion of the fatty acids to methyl esters. To detect amino sugars, the hydrolyzed samples were re-*N*-acetylated (methanol: pyridine: acetic anhydrate; 2:1:1 vol. at 100 °C; 1 h) and all methyl glycosides and methyl esters were finally converted into TMS-methyl glycosides and hydroxyl-FAMES (Fatty Acid Methyl Esters) into TMS-FAMES by using Tri-Sil HTP (Thermo) reagent for 30 min at 80 °C. The TMS derivatives were analyzed by GC-MS on a Hewlett-Packard HP5890 gas chromatograph equipped with a mass selective detector 5970 MSD using EC-1 fused silica capillary column (30m × 0.25 mm I.D.), and temperature program at 80 °C for 2 min, then ramped to 160 °C at 20 °C/min with a 2 min hold, and to 200 °C at 2 °C/min followed by an increase to 300 °C at 10 °C/min with a 20 min hold. The fatty acid identity was assigned based on the unique electron ionization (EI)-MS fragmentation patterns of the FAME of straight-chain (saturated and unsaturated) fatty acids and the EI-MS fragments of the FAME-TMS of hydroxylated fatty acids. 

### 2.12. DOC-PAGE Analysis of Bacterial and Phage Extracts

The CAP3 and LH6 aqueous, interphase, and organic extract phases were resolved by PAGE by using 18% acrylamide and deoxycholic acid (DOC) detergent [25]. The gels were stained with silver using the Bio-Rad Silver Staining Kit (Bio-Rad, Hercules, CA, USA). In addition, the gels were stained with Alcian blue dye [26], followed by the same steps as with standard silver staining. 

## 3. Results

### 3.1. Lytic Bacteriophage Isolation and Characterization

**Figure 2:**
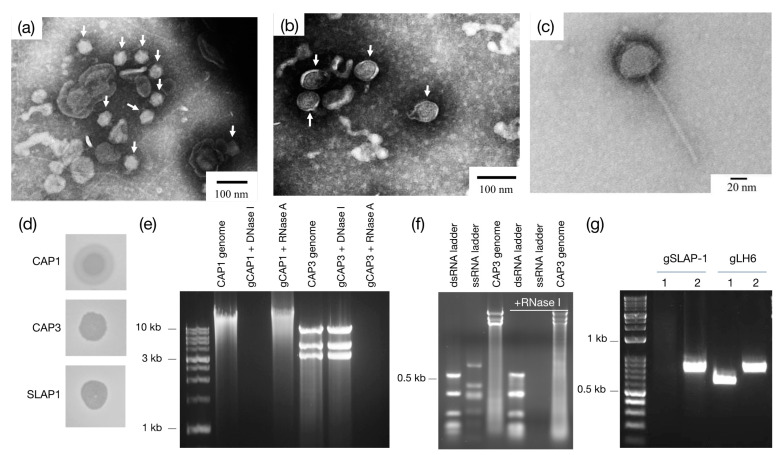
Characterization of LH6-infecting phages. Transmission electron micrographs of (**a**) CAP1, (**b**) CAP3 and (**c**) SLAP1. (**d**) Plaque morphology of isolated phages. (**e**) CAP1 and CAP3 genome before and after treatment with DNase I or RNase A. (**f**) CAP3 genome is not degraded with RNase I_f_. (**g**) PCR analysis of SLAP1 and LH6 gDNA using capsid primers from prophage 1 (lanes 1) and prophage 2 (lanes 2) in the LH6 genome [6].

Following the isolation of *A. radioresistens* strain LH6 from laying hens, feces from turkeys, ducks and laying hens were screened for phages that could infect the isolate. In total, we obtained phages from seven plaques, naming them sequentially from CAP1 to CAP7. CAP1 and CAP2 were isolated from laying hen, CAP3 from duck, and CAP4–CAP7 from turkey feces. CAP1 and CAP2 displayed a differing plaque morphology in comparison with CAP3–CAP7, showing “halo” and “non-halo” plaques, respectively (Figure 2d). All phages were unable to infect the other isolated strains of *A. radioresistens, Acinetobacter lwoffii*, or *Acinetobacter johnsonii* [7], or the *A. baumannii* laboratory strains ATCC 19606 and ATCC 17978 (results not shown). 

CAP1 and CAP3 were selected as representative phages for further characterization, based on plaque morphology. Transmission electron microscopy (TEM) of CAP1 identified ~50 nm icosahedral phages with short tails adhering to vesicles (Figure 2a, white arrows), while CAP3 was a tailless ~70 nm phage (Figure 2b, white arrows). After isolation and comparison of their genomes, it was determined that CAP1 has a DNA-based monopartite genome >10 kb, while CAP3 has a tripartite RNA-based genome with chromosomes approximately 3 kb, 4 kb and 8 kb in length (Figure 2e). Chromosomal analysis of the remaining 5 phages revealed that CAP2 has a similar DNA-based genome as CAP1, while CAP4–7 were all segmented RNA phages similar to CAP3 (results not shown). To determine if the CAP3 RNA genome was single or double stranded, RNase I_f_ was incubated with a dsRNA ladder, a ssRNA ladder, and an aliquot of CAP3 genomic RNA. Agarose gel electrophoresis showed that after incubation with RNase I_f_, only the ssRNA ladder was digested, while the dsRNA ladder and CAP3 genome were left intact (Figure 2f). This indicates that the CAP3 genome is comprised of dsRNA segments consistent with known cystoviruses.

### 3.2. Lysogenic Bacteriophage Isolation and Characterization

Whole genome sequencing identified at least two integrated prophages in the genome of *A. radioresistens* LH6 [6]. For the induction of prophages, mitomycin C was added to the culture media at a concentration of 1 µg/mL. The bacterial plaquing assay shown in Figure 1d illustrates the successful propagation and isolation of at least one of the proposed LH6 prophages, which was designated SLAP1. A titer could not be determined for this phage since it does not show the typical plaques used for PFU/mL determination of lytic phages, but instead forms a hazy zone of clearing which diminishes with decreasing concentrations of phage. Plaque assays showed that SLAP1, similar to the CAP phages, was not able to infect any of the other *A. radioresistens* or *A. baumannii* strains (data not shown). TEM images of SLAP1 (Figure 2c) show the lysogen has the typical appearance of a *Siphoviridae* phage. Measurements of the phage head resulted in an average head diameter of 50 nm, attached to a long tail with the characteristic horizontal ridges. To determine which prophage was isolated, primers were designed to probe specifically for the putative capsid genes of the two integrated prophages identified by genome sequencing of LH6 [6]. Both capsid genes were amplified from the genome of LH6, confirming their presence in the host strain, while the SLAP1 DNA only shows a PCR product for the capsid gene of prophage 2 (Figure 2g). The absence of capsid 1 in the SLAP1 genome indicates that only prophage 2 could be induced by the mitomycin C treatment.

### 3.3. LH6 Mutagenesis and Binding Target Determination

**Figure 3:**
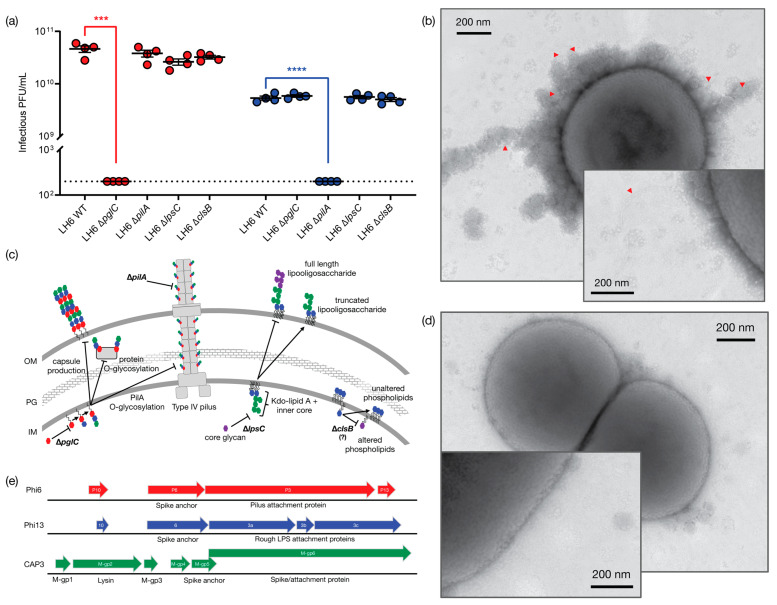
Determination of host binding receptor for CAP1 and CAP3. (**a**) Determination of plaquing efficiency for CAP1 (red circles) and CAP3 (blue circles). Significance was calculated using unpaired t-tests. *** *p* ≤ 0.001; **** *p* ≤ 0.0001 (**b**) Transmission electron micrograph of LH6 WT showing CAP3 attachment to pili (indicated by red arrows). Inset: enlargement of single pilus. (**c**) Schematic of effects on LH6 structures when *pglC*, *pilA*, *lpsC* and *clsB* genes are deleted. (**d**) Transmission electron micrograph of LH6 ∆*pilA* showing a lack of pili and reduced CAP3 association. Inset: enlargement of cell surface showing no observable pili. (**e**) Syntenic comparisons of M genomic RNA segments from Phi6, Phi13 and CAP3 showing differences and similarities in attachment proteins. Genes are drawn to scale and correspond to Figure S3 (S and L segments are shown in Figures S2 and S4, respectively).

We next sought to determine the binding target of the CAP1 and CAP3 phages. To do this, we constructed targeted deletions in the LH6 genome by replacing the gene homologs of *pglC* (initiating glycosyltransferase for O-glycans and CPS), *pilA* (pilin protein subunit), *lpsC* (LOS core glycosyltransferase) and *clsB* (cardiolipin synthase B or trehalose-phospholipid biosynthesis) [27] with an *Acinetobacter*-compatible kanamycin cassette. Although LH6 can readily take up plasmids with compatible origins of replication, such as pBAV1K-T5-*gfp* [7] and pAT4, it required the induction of an exogenous plasmid-encoded RecAB to undergo homologous recombination with the *pglC* pGEM knockout construct since electroporation without induction of RecAB yielded no transformants after repeated attempts. Therefore, all LH6 mutants were created using the recombineering plasmid expressing RecAB prior to transformation to facilitate homologous recombination as described for *A. baumannii* [24]. PCR was used to confirm that the appropriate genes were replaced with the kanamycin cassette (Figure S1). Phage plaquing assays were then performed and demonstrated that CAP1 plaquing is lowered to below the limit of detection (200 PFU/mL; dashed line) when spotted on LH6 ∆*pglC* (Figure 3a). CAP3 plaquing is similarly abolished when spotted on LH6 ∆*pilA*. Similar levels of plaquing are observed when both phages are spotted on LH6 ∆*lpsC* and ∆*clsB*. These findings indicate that the O-glycans/CPS and type IV pili are the binding targets for CAP1 and CAP3, respectively.

### 3.4. Whole Genome Sequencing of CAP3–CAP7

**Figure 4:**
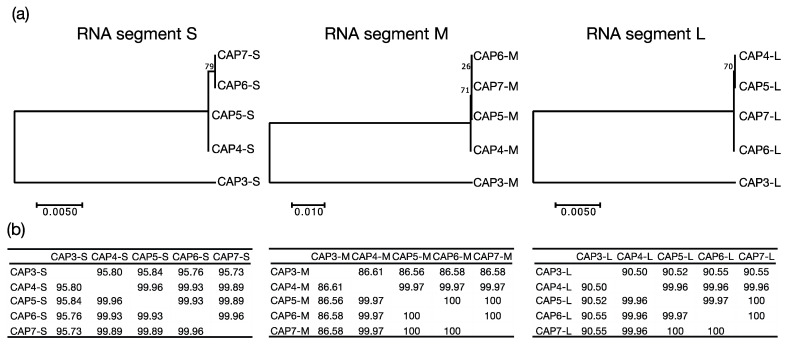
Genomic sequence comparison of CAP3–CAP7 phages. (**a**) Molecular phylogenetic analysis was determined by the Maximum Likelihood method. Each segment (S, M, L) was compared independently, and the trees with the highest likelihood are shown. Trees are drawn to scale, with branch lengths measured in the number of substitutions per site, and bootstrap values are indicated at the appropriate branch points. (**b**) Percent similarity matrices compare each RNA segment (S, M, L) to the other CAP phage RNA segments.

Following the isolation of CAP3–CAP7 and identification of their genomic contents as dsRNA, we sequenced the RNA genomes of these phages. Genome alignment and comparison of the segments revealed that CAP3 represented a distinct phage from CAP4–7 (Figure 4a). The differences between CAP4–7 were minimal, but phylogenetic analysis suggests that CAP4 and CAP5 are more closely related to each other, and CAP6 and CAP7 are essentially the same, with only minor differences in the S segment (Figure 4b). Basic local alignment search tool (BLAST) analysis revealed no significant nucleotide similarity to any genes in the NCBI database. Gene annotation software, however, annotated some genes as having domain homologies on each segment (Tables S2–S4). 

**Figure 5:**
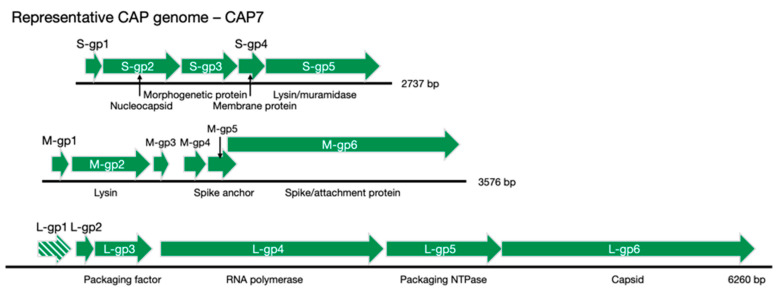
Representative CAP genome schematic. CAP7 was used as a representative genome for illustrative purposes. The L-gp1 open reading frame (ORF) is absent in the CAP3-L RNA segment (denoted by the striped arrow). Putative protein functions are listed below each gene when a function could confidently be assigned, based on a combination of synteny with other sequenced *Pseudomonas* cystovirus genomes, annotation, functional characterization, or protein modeling (RAST, Phyre2 and HHpred were used). The gene segments are drawn to scale.

**Figure 6:**
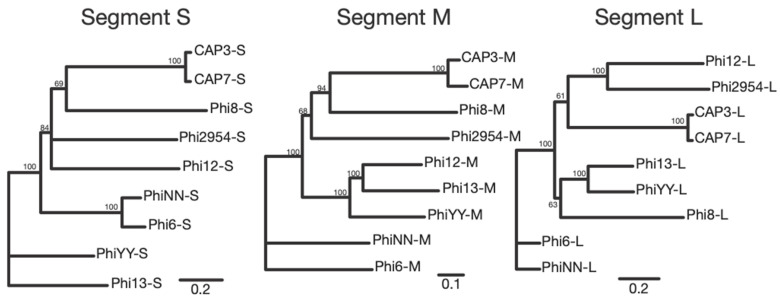
RNA segment comparison between CAP3, CAP7 and the previously sequenced *Pseudomonas*-infecting cystoviruses. Trees are drawn to scale, with branch lengths measured in the number of substitutions per site, and bootstrap values are indicated at the appropriate branch points.

Some genes had synteny to other cystovirus genomes (Figure 3c and Figures S2–S4) and could have putative functions assigned, such as L-gp3 (packaging factor), L-gp4 (RNA polymerase), L-gp5 (packaging NTPase), L-gp6 (capsid protein) and M-gp6 (spike/attachment protein). A full list of CAP cystovirus phage genes can be seen in Table 1, along with their putative functions and justifications for those assignments, where applicable. CAP7 genes can be seen in their relative positions in the segmented genome in Figure 5. Genomic comparisons were made between CAP3 and CAP7, two representative CAP phages (there is little difference between CAP4–CAP7), and the other sequenced cystovirus phages, all infecting *Pseudomonas* spp. These comparisons revealed an expected similarity between CAP3 and CAP7, which were distinct from the other cystoviruses. The large disparities between the previously sequenced cystoviruses make a lineage difficult to determine, but comparison does show that the CAP phages are most similar to *Pseudomonas* phages Phi8, Phi2954 and Phi12, depending on the RNA segment being compared (Figure 6). 

### 3.5. Glycan Analysis of Cystovirus CAP3

**Figure 7:**
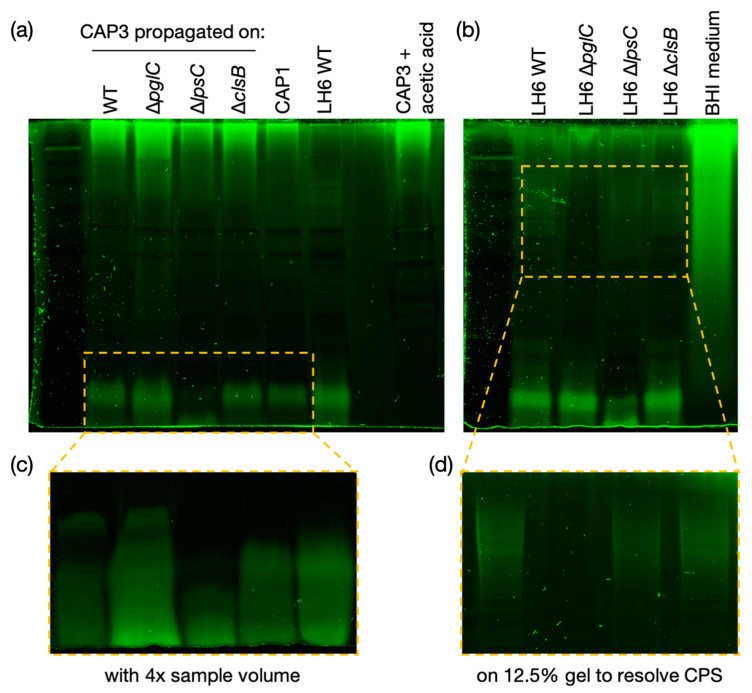
Pro-Q™ Emerald 300 staining of CAP3 glycans. (**a**) Proteinase K digests of CAP3 propagated on LH6 WT, ∆*pglC*, ∆*lpsC* and ∆*clsB*, and CAP1 phages were separated on a 15% SDS-PAGE gel and stained with Pro-Q™ Emerald 300 glycan staining kit (compare with Figure S5). CAP3 was treated with 1% acetic acid to test whether the low molecular weight glycan is a lipid-linked glycoconjugate (and Figure S6). (**b**) Proteinase K digests of LH6 WT, ∆*pglC*, ∆*lpsC* and ∆*clsB* were separated on a 15% SDS-PAGE gel and stained to show differences in glycosylation. BHI medium was also tested to determine the origin of the high molecular weight glycan in the phage samples. (**c**) CAP3 propagated on LH6 WT, ∆*pglC*, ∆*lpsC* and ∆*clsB*, and CAP1 phages were loaded with 4x volume (from Figure S6). (**d**) Proteinase K digests of LH6 WT, ∆*pglC*, ∆*lpsC* and ∆*clsB* were separated on a 12.5% SDS-PAGE gel to better resolve high molecular weight capsular polysaccharides (from Figure S7).

**Figure 8:**
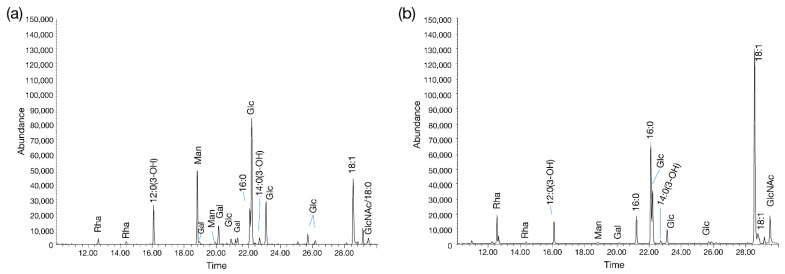
GC-MS chromatograms of CAP3 and LH6 extracts. (**a**) Chromatogram of CAP3 and (**b**) LH6 aqueous phase extracts (from Figure S8).

Following the classification of CAP3–7 in the phage family *Cystoviridae,* we were interested in determining whether any host glycan intermediates were associated with the phages, and used CAP3 as the representative. CAP3 was purified to a high titer (>1 × 10^10^ PFU/mL), using PEG 8000 precipitation and CsCl gradient ultracentrifugation. Purified CAP3 lysates were separated by SDS-PAGE followed by Pro-Q™ Emerald 300 staining for detection of glycan structures (Figure 7). Two regions showed staining, a high molecular weight (MW) glycan above 245 kDa, and a lower MW glycoconjugate in the region of 11-17 kDa (Coomassie stained version of Figure 7 in Figure S5 with molecular weights). It was determined that the high MW glycan was a contaminant from the BHI medium used to propagate the phage (Figure 7b). To further characterize the unknown low MW glycans, CAP3 was propagated on LH6 WT, ∆*pglC*, ∆*lpsC* and ∆*clsB* mutants, or the phage was treated with 1% acetic acid, and then lysates were separated by SDS-PAGE and stained. Phage treatment with acetic acid caused loss of the low MW glycan staining on the gel (Figure 7a), indicating that this structure is a lipid-linked glycoconjugate. There was no effect on the low MW glycan after phage propagation on the ∆*pglC* mutant (Figure 7 and Figure S6). This indicated that the glycans are not derived from CPS/glycoproteins and is consistent with the finding that the phage low MW stained material did not correspond to the CPS mass that was lost in the ∆*pglC* mutant (Figure 7d and Figure S7), and that proteinase K treatment did not alter the staining patterns (which would disappear if glycoproteins were involved). There was, however, a downward shift of CAP3 low MW staining after propagation on LH6 ∆*lpsC*, which mirrored the shift observed with LH6 ∆*lpsC* resulting from the truncation of the LOS core oligosaccharide. The presence of LOS in both samples was supported by the GC-MS findings (Figure 8 and Figure S8) where diagnostic hydroxylated fatty acids (12:0[3-OH] and 14:0[3-OH]) were detected together with possible glucosamine residues (would be converted to GlcNAc after *N*-acetylation), along with other glycans, and longer chain fatty acids likely from phospholipids (Figure 8 and Figure S8). However, although the buffer control shows that the LOS observed in the CAP3 gellanes is not due to contamination from the buffer, the purification of the lytic CAP1 phage, using the same method, suggests host LOS is introduced through the phage purification process, albeit at somewhat reduced levels (Figure 7a). An additional glycan-staining band is visible in varying concentrations just above the LOS in the CAP3 preparations, That is not present after CAP1 purification (particularly visible when higher phage concentrations are loaded) (Figure 7c). This band may correspond with the unique Alcian blue staining band visible in the CAP3 aqueous phase (Figure S9, grey arrow).

## 4. Discussion

Bacteriophages are predicted to infect every bacterial genus, but scientists have only examined the tip of the iceberg when exploring the diversity of phages that are all around us. In this work, we describe the isolation and characterization of eight phages infecting the multidrug resistant *A. radioresistens* strain LH6. CAP1 and CAP2 were isolated from chicken feces, while CAP3 was from duck feces, and CAP4–7 were isolated from turkey feces. Finding phages infecting LH6 that were isolated from the same chicken feces, is not surprising, however, the isolation of CAP3–7 from different birds was unexpected. MEGA7 phylogenetic analysis grouped CAP3 apart from CAP4–CAP7 (Figure 3), which is suggestive of a persistence in the duck-husbandry environment, separate from the turkeys that CAP4–CAP7 were derived from. The extremely high sequence similarity of the CAP4–CAP7 phages suggests that they are likely the same phage, or variants of the same phage. The background rate of mutation and genetic drift for these phages is unknown, but this information could yield insight into how recently the phages shared an ancestor. Our results suggest that either a compatible strain of *A. radioresistens* was present in those birds, which the CAP phages could infect and replicate in, or these phages have a high environmental persistence in a poultry-husbandry setting.

The different plaque morphologies of the isolated phages are also of interest. CAP1 and CAP2 both have a “halo” phenotype surrounding the observed plaques (Figure 2d). This can be explained by diffusion of a phage-associated glycolytic enzyme, and is consistent with other bacteriophages [28], including *Salmonella*-infecting phage P22 which enzymatically degrades the *Salmonella* O-antigen polysaccharide [29]. CAP3–7, however, have a different plaque morphology which represents the more classic “pinprick” type plaque with a defined zone of clearing. SLAP1 is different from either of the other plaque morphologies. The zone of clearing produced by spotting the phage onto LH6 is semi-transparent, probably due to the lysogenic nature of the phage which allows for the growth of LH6 even in the presence of high phage concentrations. Interestingly, the phage can only be visualized by plaquing assays in the presence of mitomycin C, without which, no zone of clearing is formed, even at high phage densities (results not shown).

After genome and TEM characterization of the CAP1–7 and SLAP1 phages, we propose that CAP1 and CAP2 belong to the *Podoviridae*. The DNA content, icosahedral heads and short tails are consistent with other members of this phage family [30]. Second, we propose that SLAP1 belongs to the *Siphoviridae* due to the icosahedral head and long flexible tail, which is the hallmark of this phage family [31]. Finally, we propose that CAP3–CAP7 belong to a new phage genus within the family *Cystoviridae.* The enveloped capsid, along with the segmented dsRNA genome are indicative of this family, along with the sequencing identity, which shares synteny and some homology with other cystoviruses infecting members of the *Pseudomonas* genus (Figure 6 and Figures S2–S4) [32,33]. Interestingly, these are the first cystovirus phages that infect any species besides the seven characterized phages that infect *P. syringae* and *P. aeruginosa* [17]. Whole genome sequencing and subsequent analysis showed that some of the CAP genes could be assigned putative functions based on synteny to the other cystoviruses (Table 1, Figure 3). The lack of distinct synteny shows that these viruses mutate at a high rate and can undergo genomic rearrangement, while maintaining a similar genome size and life cycle. Better understanding of these processes could give insight into the evolutionary tendencies of RNA viruses as whole.

After identifying CAP3–7 as cystoviruses, we were interested in investigating whether or not their envelopes, derived from the host’s inner membrane [32], contained any glycan intermediates known to be synthesized at this location. To test this, we performed several glycan stains on CAP3 lysates and observed two areas of SDS-PAGE staining corresponding to a high MW glycan and a low MW glycan. We determined that the high MW stained material was a contaminant from the growth medium while the lower MW structure is a lipid-linked glycan, presumably an oligosaccharide attached to lipid A (i.e., LOS) in *A. radioresistens*. Using mutants disrupting CPS/O-glycan, LOS core and cardiolipin (or trehalose phospholipid [34]) biosynthesis, we were able to determine that the low MW glycan is not a precursor to CPS, and was similarly unaffected in the ∆*clsB* mutant strain and derived-phage samples. The glycoconjugate was, however, truncated after the LH6 mutation of *lpsC* and subsequent phage propagation on that strain. LpsC is homologous to conserved LOS core β-glucosyltransferases found in most Gram-negative bacteria [35]. Inactivation of the enzyme leads to LOS truncation, consistent with the faster LOS migration observed by SDS-PAGE for both LH6 ∆*lpsC* and CAP3 propagated on this mutant. Similarly, mass-spectrometry analyses of both LH6 and purified CAP3 showed signals corresponding to β-hydroxy fatty acids unique to bacterial lipid A (Figure 8). However, it remains to be determined whether cystoviruses have glycoconjugates (i.e., host lipooligosaccharides) embedded in their membranes or whether our observations are an artifact of phage propagation on the host.

It is difficult to imagine a scenario where host glycan intermediates would not be present during phage induced membrane-budding, but cystovirus recruitment of host membranes appears to exclude host proteins [36], so it is possible that only phospholipids are selectively recruited from the host. Laurinavičius et al. demonstrated that the Phi6 phage had a similar phospholipid content as its host’s inner membrane, and our GC-MS profiles also detect similar fatty acid compositions (16:0 and 18:1) [37]. However, the researchers used organic extraction to intentionally isolate phospholipids while we observed that most CAP3 membrane components partitioned into the aqueous phase. Similar studies need to be done to determine the full composition of the LH6 inner membrane versus the LH6 host-derived membrane. In the Laurinavičius et al. study, the predominant lipids were phosphatidylethanolamine and phosphatidylglycerol, along with low levels of cardiolipin [37]. Recent studies in *Salmonella* have identified a new class of glycolipids, 6-phosphatidyltrehalose and 6,6-diphosphatidyltrehalose, that require the cardiolipin synthase (ClsB) for synthesis [34]. Since we recently demonstrated that *A. radioresistens* LH6 is capable of synthesizing very high levels of trehalose [37], we mutated its *clsB* homolog to see if any changes were observed in the CAP3 glycan staining profile (particularly since GC-MS analyses identified high levels of Glc in the CAP3 chromatogram, Figure 8a), but again, a more thorough membrane compositional analysis of both LH6 and CAP3 needs to be done at a larger scale to confirm which glycoconjugate structures are actually present in the membrane; characterization of phage particles by immunogold labeling is currently underway to prove if any of these structures are phage-associated.

Additionally, we were interested in identifying the phage receptors necessary for host recognition. Based on the halo plaque morphology observed for CAP1, we predicted this phage would interact with a surface polysaccharide, and since Acinetobacters, including other strains of *A. radioresistens* [38], typically express rough LPS (i.e., LOS), we wanted to target the CPS. We identified several putative CPS transport and biosynthesis genes including *pglC*, which is predicted to encode an enzyme catalyzing the addition of di-N-acetylbacillosamine (diNAcBac) or GlcNAc to the lipid carrier UndP based on gene homology and synteny with the related *A. baumannii* [14,39,40]. We presume that the O-linked glycosylation pathway is similarly impeded (Figure 3b), but have not yet confirmed this experimentally. We then tested the ability of the representative phages CAP1 and CAP3 to plaque on the ∆*pglC* mutant (Figure 3a) and as predicted, CAP1 was unable to plaque on this mutant. We next sought to identify the host receptor for CAP3. The best-characterized member of the cystovirus family, Phi6, binds to the pilus of *P. syringae* [41], while other cystovirus phages bind to LPS. The high sequence synteny of the CAP3 M segment to the pilin-binding phage Phi6, is suggestive that the M-gp6 gene encodes a protein that will form a trimeric spike protein mediating host attachment. Furthermore, in other cystoviruses that do not bind pilin, but instead target LPS, the spike homolog is split into three ORFs that form a heterotrimer to bind *Pseudomonas* LPS (Figure 3c, Figure S3). We thus hypothesized that CAP3 would require PilA and this was supported by our experiments demonstrating that CAP3 did not plaque on the LH6 ∆*pilA* mutant, indicating that CAP3 requires the structural component of the LH6 pilus. Interestingly, it was recently shown that when the *P. syringae pilA* gene was expressed in a *P. aeruginosa* ∆*pilA* mutant, both the *P. syringae* phage Phi6 and the *P. aeruginosa* phage PO4 were capable of infecting *P. aeruginosa* [42], suggesting that cystoviruses may be engineered to acquire a broader host range that extends beyond the strain and species level. These experiments represent the first step in understanding and potentially exploiting these *Acinetobacter* viruses to recognize and infect other notorious *Acinetobacter* species, which result in some of the most deadly and difficult to treat bacterial infections in the world.

## 5. Conclusions

The accumulation of knowledge centered on bacteriophage diversity, life cycles and binding targets are important foundations to better understand these viruses and exploit their use by researchers and physicians combatting drug resistant infections with phage therapy. We have isolated several new bacteriophages infecting the multidrug resistant strain LH6, five of which belong to a new phage genus in the family *Cystoviridae*. Furthermore, these phages may possess surface glycans, an observation worth exploring, particularly since this would have implications on how this family of phages may interact with the eukaryotic hosts that the propagating microbes colonize. These exciting findings expand the field of bacteriophage diversity and structure, and further studies will provide a better understanding of the life cycle of these rare bacteriophages. 

## Figures and Tables

**Table 1:** CAP cystovirus ORFs with genetic annotations, putative protein functions, and *Pseudomonas* phage Phi6 homologs.

Segment-ORF	Protein Annotation	Phi6 Homolog	Putative Function	Justification
S-gp1	Hypothetical protein	N/A	N/A	N/A
S-gp2	Hypothetical protein	P8	Nucleocapsid	Protein homology & synteny
S-gp3	Zinc finger protein 208	P12	Morphogenetic protein	Protein analysis & synteny
S-gp4	Cysteine methyltransferase	P9	Membrane protein	Synteny
S-gp5	Hypothetical protein	P5	Lysin/Muramidase	Protein analysis & synteny
M-gp1	Hypothetical protein	N/A	N/A	N/A
M-gp2	SpoIIE-like protein phosphate domain protein	N/A	Lysin	Protein analysis
M-gp3	Hypothetical protein	N/A	N/A	N/A
M-gp4	Hypothetical protein	N/A	N/A	N/A
M-gp5	Hypothetical protein	P6	Spike anchor	Protein analysis & synteny
M-gp6	Amidohydrolase	P3	Spike/attachment protein	Synteny
L-gp1	Hypothetical protein	N/A	N/A	N/A
L-gp2	Hypothetical protein	N/A	N/A	N/A
L-gp3	Hypothetical protein	P7	Packaging factor	Synteny
L-gp4	RNA polymerase	P2	RNA polymerase	Annotation & synteny
L-gp5	AAA family ATPase	P4	Packaging NTPase	Annotation & synteny
L-gp6	Hypothetical protein	P1	Capsid	Synteny

## Data Availability

All sequencing data has been deposited into NCBI and will be released publicly once the manuscript is accepted for publication with the following accession numbers: CAP3-L: MZ558504; CAP3-M: MZ558505; CAP3-S: MZ558506; CAP4-L: MZ558507; CAP4-M: MZ558508; CAP4-S: MZ558509; CAP5-L: MZ558510; CAP5-M: MZ558511; CAP5-S: MZ558512; CAP6-L: MZ558513; CAP6-M: MZ558514; CAP6-S: MZ558515; CAP7-L: MZ558516; CAP7-M: MZ558517; CAP7-S: MZ558518. All other data is available within the manuscript and Supplemental Materials.

## Supplementary Materials

The following are available online at https://www.mdpi.com/article/10.3390/v13081652/s1, Figure S1: PCR confirmation of LH6 mutagenesis in genes *pglC, pilA, lpsC*, and *clsB*, Figure S2: Alignments of CAP3 and CAP7 with *Pseudomonas*-infecting cystovirus S segments for syntenic comparisons, Figure S3: Alignments of CAP3 and CAP7 with *Pseudomonas*-infecting cystovirus M segments for syntenic comparisons, Figure S4: Alignments of CAP3 and CAP7 with *Pseudomonas*-infecting cystovirus L segments for syntenic comparisons, Figure S5: Coomassie stained 15% SDS-PAGE of lysates from *A. radioresistens* LH6 cells and purified CAP1 and CAP3 phages corresponding to proteinase K treated lysates seen in Figure 7a, Figure S6: Coomassie and Pro-Q™ Emerald 300 stained gels of CAP3 and CAP1 lysates, Figure S7: Coomassie and Pro-Q™ Emerald 300 stained gels of LH6 lysates, Figure S8: All GC-MS chromatograms corresponding to the experiment represented in Figure 8, and Table S1: Primers used in this study, Table S2: Small RNA segment open reading frames for CAP3-CAP7 phages, Table S3: Medium RNA segment open reading frames for CAP3–CAP7 phages, and Figure S9: 18% DOC-PAGE analysis of bacterial and phage extracts examined by GC-MS in Figure S8 (and Figure 8). Table S4: Large RNA segment open reading frames for CAP3–CAP7 phages.
