# Supplementary material for: RNA and Sugars, Unique Properties of Bacteriophages Infecting Multidrug Resistant Acinetobacter radioresistens Strain LH6
Source: Viruses. 2021 Aug 20;13(8):1652. doi: 10.3390/v13081652 (PMC8402811; doi:10.3390/v13081652)
Supplement: Supplementary file 1 [file viruses-13-01652-s001.zip › viruses-1324955-supplementary.pdf]

RNA and Sugars, Unique Properties of Bacteriophages Infecting Multidrug Resistant *Acinetobacter radioresistens* Strain LH6

Clay S. Crippen [1,2], Bibi Zhou [1,2], Silke Andresen [1,2], Robert T. Patry[1,2#], Artur Muszyński [2], Craig T. Parker [3], Kerry K. Cooper [4] and Christine M. Szymanski[1,2*]

Contained:
Supplementary Tables S1–S4
Supplementary Figures S1–S9

Table S1. Primers used in this study.

Oligo	Sequence 5′ to 3′	Use
pP1capsid-F	GCTTTTCAATTATGCAGATCAGC	Identifying SLAP1 prophage
pP1capsid-R	GTTTTACCCAGATTGTCATGAATG	Identifying SLAP1 prophage
pP2capsid-F	TGAATGAGCTTAAAAGCCGTG	Identifying SLAP1 prophage
pP2capsid-R	AGAATTTATTAGCGGCAATGGC	Identifying SLAP1 prophage
pBAV1K KAN-F	GACGTCAAATTCTATCATAAT	PCR kan cassette
pBAV1K KAN-R	CTAAAACAATTCATCCAGTAA	PCR kan cassette
ApaI-KO *pglC* upstream-F	GCAGGGCCCATGAAATTCTTAATTATTGCTAGC	PCR *pglC* construct upstream
SphI-KO *pglC* upstream-R	GCAGCATGCTTAGATTAAACCCATTTCTTGCATC	PCR *pglC* construct upstream
SpeI-RBS-KO *pglC* downstream-F	GCAACTAGTAAGAAGGAGATATACATGACCAGTAATTCTGTTTATGCG	PCR *pglC* construct downstream
PstI-KO *pglc* downstream-R	GCACTGCAGAGGATTAGCACCGGCTGTGACAATTG	PCR *pglC* construct downstream
ApaI-KO *pilA* upstream-F	GCAGGGCCCGGGATTGATGATAGATTTAGAAATTAAGAAATC	PCR *pilA* construct upstream
NcoI-KO *pilA* upstream-R	GCACCATGGGACGTCAAATTCTATCATAATTG	PCR *pilA* construct upstream
KO *pilA* upstream with kan pro-F	AATTTTTCTTGGCATTTATTATTTCCTTCCTCTTTTCTAC	PCR *pilA* construct upstream
KO *pilA* upstream with kan pro-R	GAAGGAAATAATAAATGCCAAGAAAAATTATACCTTACC	PCR *pilA* construct upstream
SalI-KO *pilA* downstream-F	GCAGTCGACAACATTCTCCACCACATGTG	PCR *pilA* construct downstream
SacI-KO *pilA* downstream-R	GCAGAGCTCAAGTGCTTCCACTGCTGGTGTG	PCR *pilA* construct downstream
ApaI-KO *lpsC* upstream-F	GCAGGGCCCAGAATTCCGGGCTTTCAGAAGCTC	PCR *lpsC* construct upstream
SphI-KO *lpsC* upstream-R	GCAGCATGCTCATAACTTATAACGTCCTTTAGC	PCR *lpsC* construct upstream
SpeI-RBS-KO *lpsC* downstream-F	GCAACTAGTAAGAAGGAGATATAC ATGAAAATTGTTCAGGTATTGGC	PCR *lpsC* construct downstream
SalI-KO *lpsC* downstream-R	GCAGTCGACTTAAGTGTTCAGCAAACCTTTATAAATATTAAG	PCR *lpsC* construct downstream
SphI-KO *clsB* upstream-F	GCAGCATGCCTTGGTCATGGTGCGTGTGATG	PCR *clsB* construct upstream
NcoI-KO *clsB* upstream-R	GCACCATGGAAAAACAGATTACATTAAATCTCG	PCR *clsB* construct upstream
SpeI-RBS-KO *clsB* downstream-F	GCAACTAGTAAGAAGGAGATATACATGAAATCACGTGCAGCAGTCGC	PCR *clsB* construct downstream
SalI-KO *clsB* downstream-R	GCAGTCGACCTAAAAATGAATCACTGTACGGATAG	PCR *clsB* construct downstream
lpsC upstream F	ATGATGTGGTTTTTATACGTTG	PCR *lpsC* knockout confirmation
lpsC downstream R	CCTTTATAAATATTAAGCGTC	PCR *lpsC* knockout confirmation
clsB upstream F	CCTGATATTCTATATAGTCCC	PCR *clsB* knockout confirmation
clsB downstream R	GATTTCTGCTACTACAGTGTATTC	PCR *clsB* knockout confirmation

Restriction sites are underlined, restriction enzymes (if applicable) are indicated.

Table S2. Small RNA segment open reading frames for CAP3-CAP7 phages.

Segment Name	ORF Name	Protein Annotation	Minimum	Maximum	Length	Direction
CAP3-S	gp1	Hypothetical protein	121	279	159	forward
CAP3-S	gp2	Hypothetical protein	283	963	681	forward
CAP3-S	gp3	Zinc finger protein 208	963	1421	459	forward
CAP3-S	gp4	Cysteine methyltransferase	1436	1675	240	forward
CAP3-S	gp5	Hypothetical protein	1675	2676	1002	forward
CAP4-S	gp1	Hypothetical protein	120	278	159	forward
CAP4-S	gp2	Hypothetical protein	282	962	681	forward
CAP4-S	gp3	Zinc finger protein 208	962	1420	459	forward
CAP4-S	gp4	Cysteine methyltransferase	1435	1674	240	forward
CAP4-S	gp5	Hypothetical protein	1674	2675	1002	forward
CAP5-S	gp1	Hypothetical protein	121	279	159	forward
CAP5-S	gp2	Hypothetical protein	283	963	681	forward
CAP5-S	gp3	Zinc finger protein 208	963	1421	459	forward
CAP5-S	gp4	Cysteine methyltransferase	1436	1675	240	forward
CAP5-S	gp5	Hypothetical protein	1675	2676	1002	forward
CAP6-S	gp1	Hypothetical protein	121	279	159	forward
CAP6-S	gp2	Hypothetical protein	283	963	681	forward
CAP6-S	gp3	Zinc finger protein 208	963	1421	459	forward
CAP6-S	gp4	Cysteine methyltransferase	1436	1675	240	forward
CAP6-S	gp5	Hypothetical protein	1675	2676	1002	forward
CAP7-S	gp1	Hypothetical protein	122	280	159	forward
CAP7-S	gp2	Hypothetical protein	284	964	681	forward
CAP7-S	gp3	Zinc finger protein 208	964	1422	459	forward
CAP7-S	gp4	Cysteine methyltransferase	1437	1676	240	forward
CAP7-S	gp5	Hypothetical protein	1676	2677	1002	forward

Table S3. Medium RNA segment open reading frames for CAP3-CAP7 phages.

Segment Name	ORF Name	Protein Annotation	Minimum	Maximum	Length	Direction
CAP3-M	gp1	Hypothetical protein	38	184	147	forward
CAP3-M	gp2	SpoIIE-like protein phosphate domain protein	213	908	696	forward
CAP3-M	gp3	Hypothetical protein	917	1048	132	forward
CAP3-M	gp4	Hypothetical protein	1177	1302	126	forward
CAP3-M	gp5	Hypothetical protein	1299	1562	264	forward
CAP3-M	gp6	Amidohydrolase	1474	3495	2022	forward
CAP4-M	gp1	Hypothetical protein	38	184	147	forward
CAP4-M	gp2	SpoIIE-like protein phosphate domain protein	213	908	696	forward
CAP4-M	gp3	Hypothetical protein	918	1049	132	forward
CAP4-M	gp4	SRPBCC family protein	1222	1383	162	forward
CAP4-M	gp5	Hypothetical protein	1380	1652	273	forward
CAP4-M	gp6	Amidohydrolase	1555	3585	2031	forward
CAP5-M	gp1	Hypothetical protein	38	184	147	forward
CAP5-M	gp2	SpoIIE-like protein phosphate domain protein	213	908	696	forward
CAP5-M	gp3	Hypothetical protein	918	1049	132	forward
CAP5-M	gp4	SRPBCC family protein	1222	1383	162	forward
CAP5-M	gp5	Hypothetical protein	1380	1652	273	forward
CAP5-M	gp6	Amidohydrolase	1555	3585	2031	forward
CAP6-M	gp1	Hypothetical protein	38	184	147	forward
CAP6-M	gp2	SpoIIE-like protein phosphate domain protein	213	908	696	forward
CAP6-M	gp3	Hypothetical protein	918	1049	132	forward
CAP6-M	gp4	SRPBCC family protein	1222	1383	162	forward
CAP6-M	gp5	Hypothetical protein	1380	1652	273	forward
CAP6-M	gp6	Amidohydrolase	1555	3585	2031	forward
CAP7-M	gp1	Hypothetical protein	38	184	147	forward
CAP7-M	gp2	SpoIIE-like protein phosphate domain protein	213	908	696	forward
CAP7-M	gp3	Hypothetical protein	918	1049	132	forward
CAP7-M	gp4	SRPBCC family protein	1222	1383	162	forward
CAP7-M	gp5	Hypothetical protein	1380	1652	273	forward
CAP7-M	gp6	Amidohydrolase	1555	3585	2031	forward

Table S4. Large RNA segment open reading frames for CAP3-CAP7 phages.

Segment Name	ORF Name	Protein Annotation	Minimum	Maximum	Length	Direction
CAP3-L	gp2	Hypothetical protein	253	420	168	forward
CAP3-L	gp3	Hypothetical protein	417	914	498	forward
CAP3-L	gp4	RNA polymerase	985	2937	1953	forward
CAP3-L	gp5	Packaging NTPase	2934	3947	1014	forward
CAP3-L	gp6	Hypothetical protein	3957	6140	2184	forward
CAP4-L	gp1	Hypothetical protein	259	558	300	forward
CAP4-L	gp2	Hypothetical protein	603	770	168	forward
CAP4-L	gp3	Hypothetical protein	767	1264	498	forward
CAP4-L	gp4	RNA polymerase	1335	3285	1951	forward
CAP4-L	gp5	Packaging NTPase	3282	4295	1014	forward
CAP4-L	gp6	Hypothetical protein	4305	6488	2184	forward
CAP5-L	gp1	Hypothetical protein	296	559	264	forward
CAP5-L	gp2	Hypothetical protein	604	771	168	forward
CAP5-L	gp3	Hypothetical protein	768	1265	498	forward
CAP5-L	gp4	RNA polymerase	1336	3288	1953	forward
CAP5-L	gp5	Packaging NTPase	3285	4298	1014	forward
CAP5-L	gp6	Hypothetical protein	4308	6491	2184	forward
CAP6-L	gp1	Hypothetical protein	272	559	288	forward
CAP6-L	gp2	Hypothetical protein	604	771	168	forward
CAP6-L	gp3	Hypothetical protein	768	1265	498	forward
CAP6-L	gp4	RNA polymerase	1336	3288	1953	forward
CAP6-L	gp5	Packaging NTPase	3285	4298	1014	forward
CAP6-L	gp6	Hypothetical protein	4308	6491	2184	forward
CAP7-L	gp1	Hypothetical protein	272	559	288	forward
CAP7-L	gp2	Hypothetical protein	604	771	168	forward
CAP7-L	gp3	Hypothetical protein	768	1265	498	forward
CAP7-L	gp4	RNA polymerase	1336	3288	1953	forward
CAP7-L	gp5	Packaging NTPase	3285	4298	1014	forward
CAP7-L	gp6	Hypothetical protein	4308	6491	2184	forward

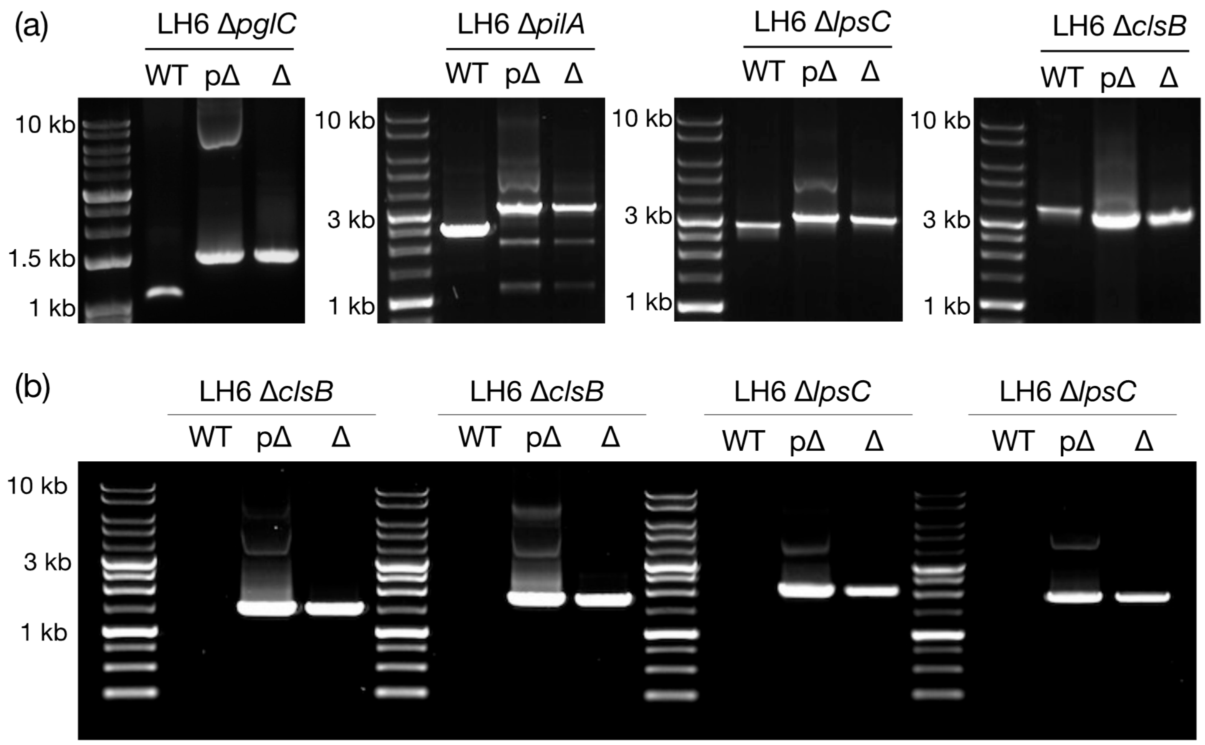

Figure S1. PCR confirmation of LH6 mutagenesis in genes *pglC, pilA, lpsC,* and *clsB*. Lane labels are as follows: WT represents PCR of LH6 wild type genomic DNA; pΔ represents PCR of knockout construct plasmid; Δ represents PCR of indicated mutant genomic DNA. (a) Products were generated using primers flanking the recombination event. (b) Products for *clsB* and *lpsC* were generated using primers inside of the insertional kanamycin cassette and in the regions of homology upstream (left of each pair) and downstream (right of each pair) of the deleted gene target as additional confirmation.

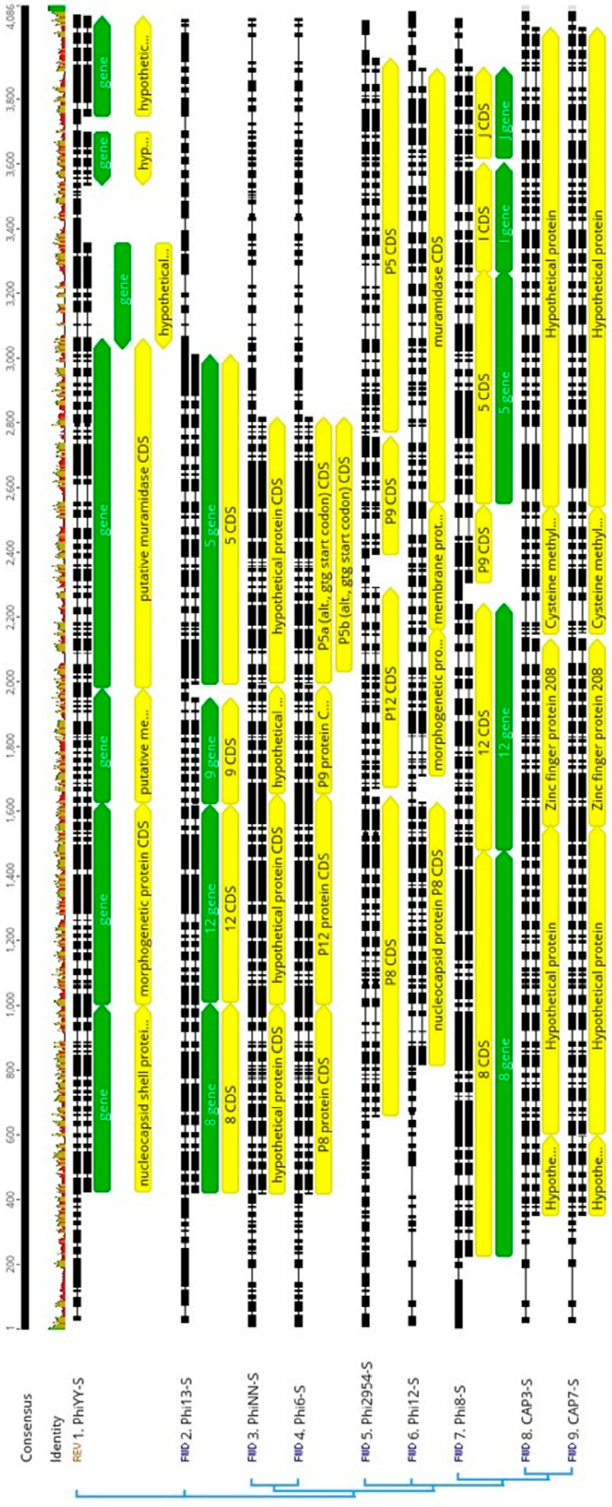

Figure S2. Alignments of CAP3 and CAP7 with *Pseudomonas* infecting cystovirus S segments for syntenic comparisons.

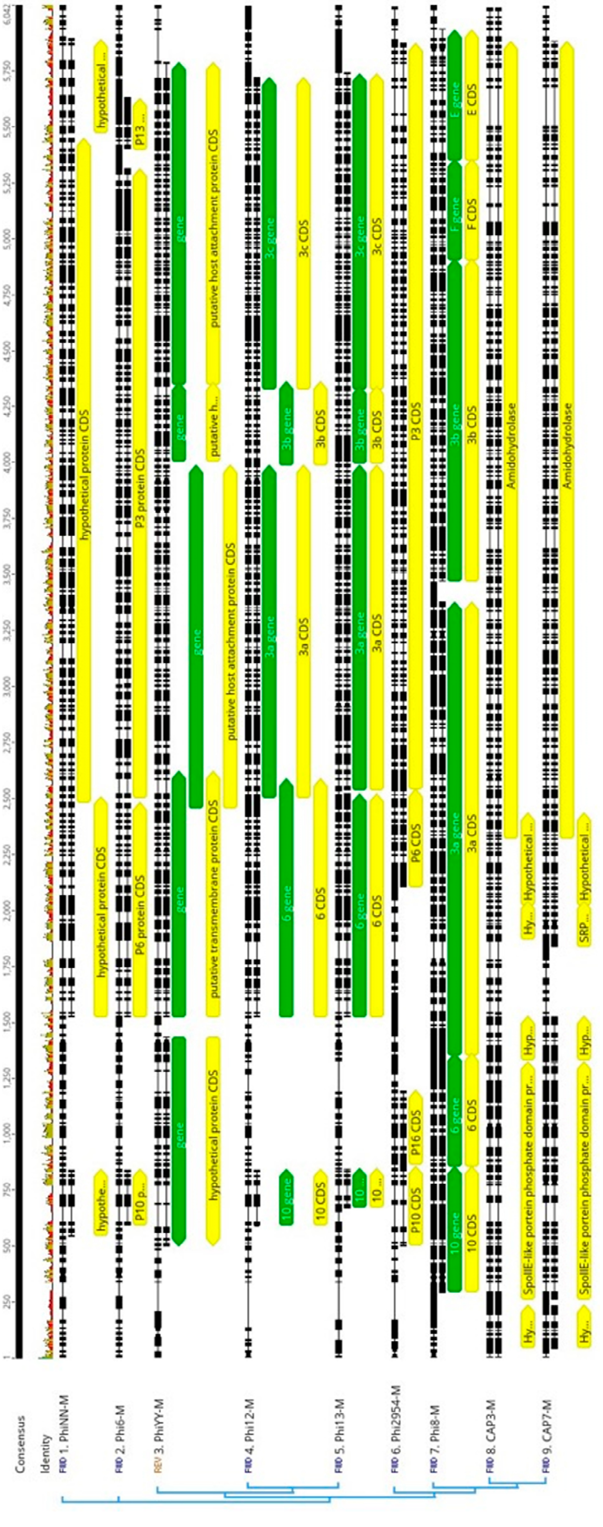

Figure S3. Alignments of CAP3 and CAP7 with *Pseudomonas* infecting cystovirus M segments for syntenic comparisons.

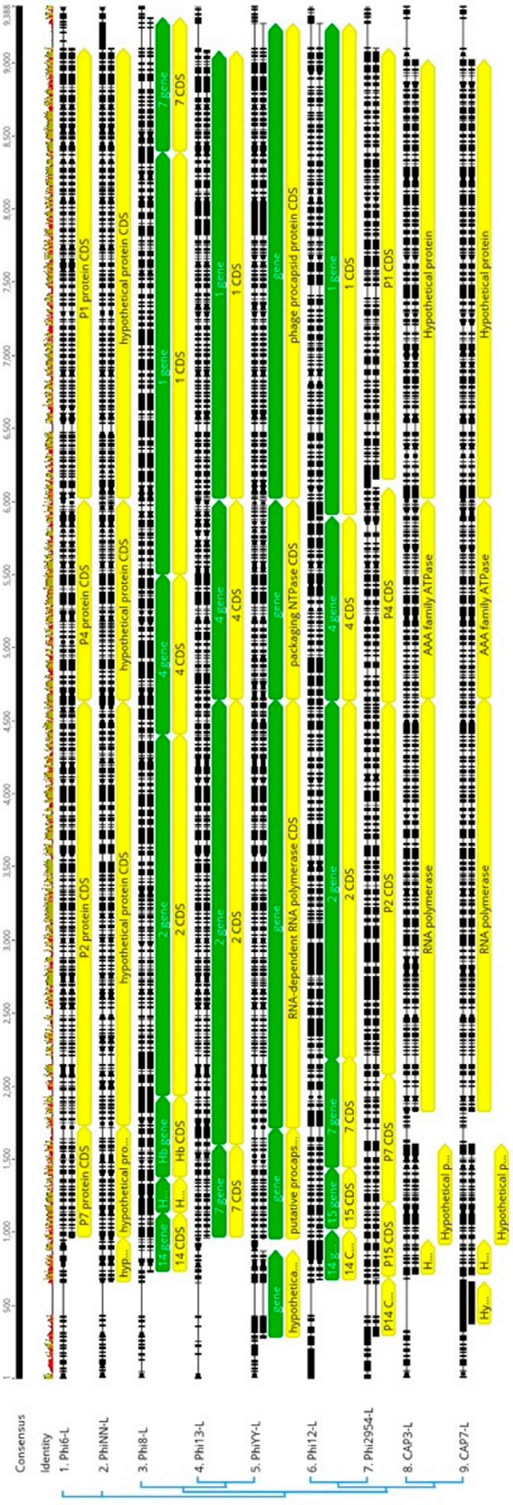

Figure S4. Alignments of CAP3 and CAP7 with *Pseudomonas* infecting cystovirus L segments for syntenic comparisons.

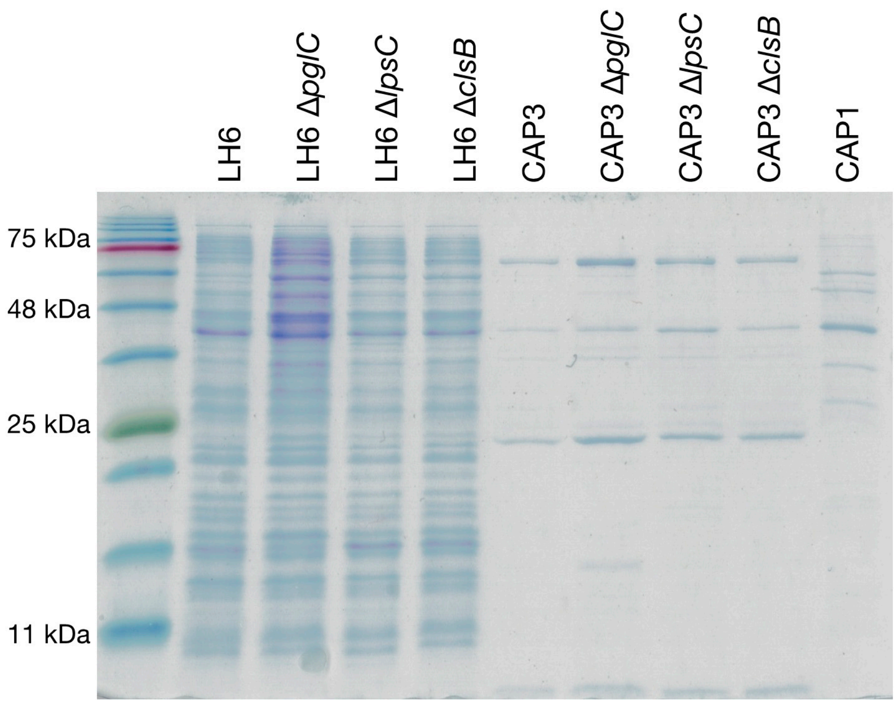

Figure S5. Coomassie stained 15% SDS-PAGE of lysates from *A. radioresistens* LH6 cells and purified CAP1 and CAP3 phages corresponding to proteinase K treated lysates seen in Figure 7a. The mutant name next to CAP3 indicates which LH6 mutant the CAP3 phage was propagated on before purification and analysis.

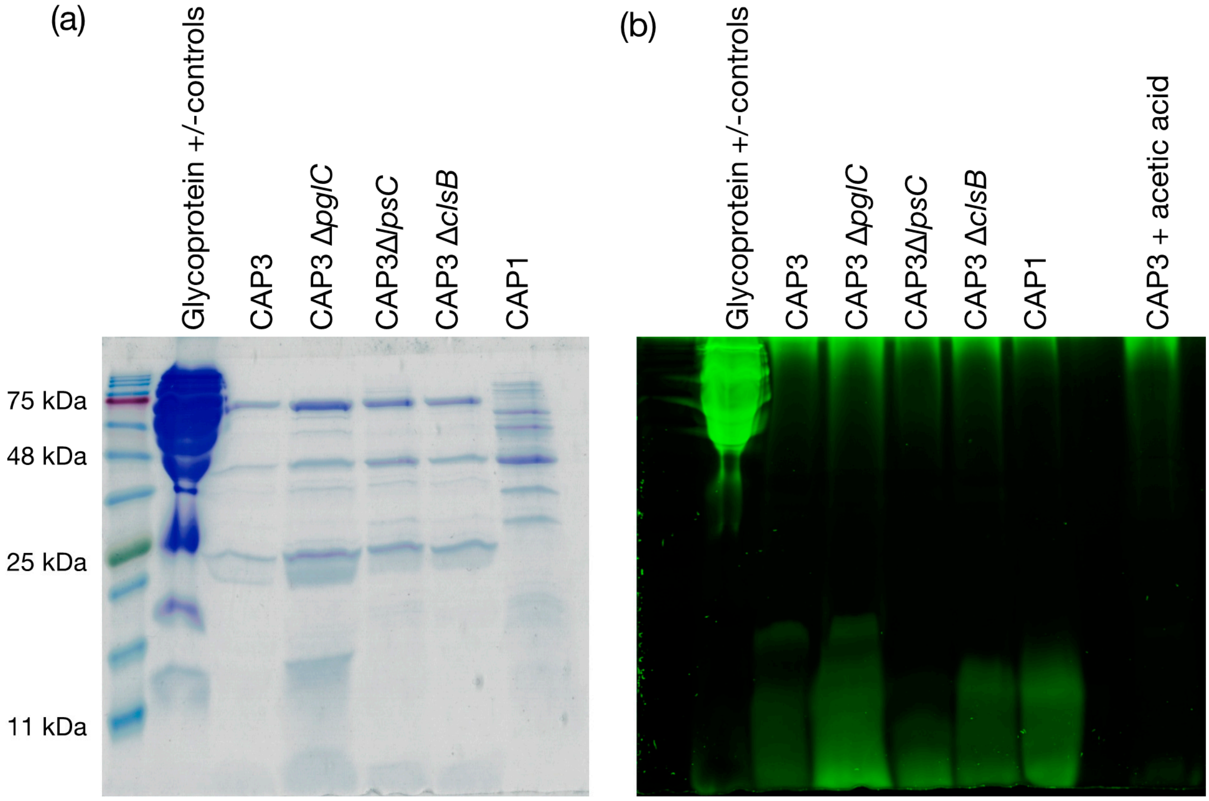

Figure S6. Coomassie and Pro-Q™ Emerald 300 stained gels of CAP3 and CAP1 lysates. (a) CAP3 and CAP1 lysates were separated on 15% SDS-PAGE and Coomassie stained. (b) Proteinase K treated CAP3 and CAP1 lysates were separated on 15% SDS-PAGE and stained for glycans (part shown in Figure 7c). A glycoprotein standard was included as part of the kit (staining controls appear as a cluster of positive glycoprotein bands between 40-80 kD and non-glycoprotein bands are only visible when stained with Coomassie) was used to confirm the activity of the glycostain. The mutant name next to CAP3 indicates which LH6 mutant the CAP3 phage was propagated on before purification and analysis. CAP3 was also treated with 1% acetic acid to determine which glycans were lipid-linked and would disappear due to disruption of the labile bond.

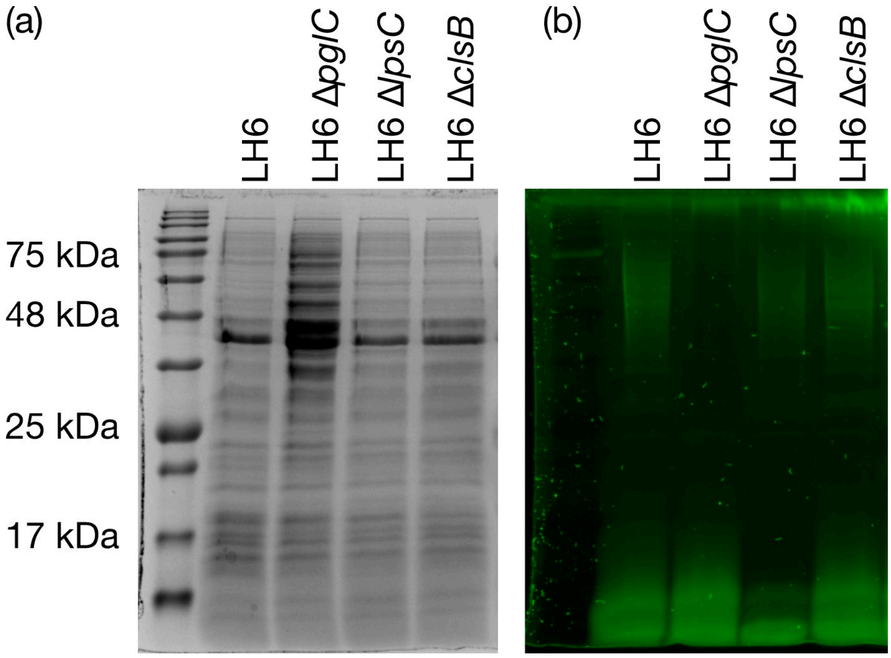

Figure S7. Coomassie and Pro-Q™ Emerald 300 stained gels of LH6 lysates. (a) Approximately equivalent amounts of LH6 WT and mutant cells were lysed, separated on a 12.5% SDS-PAGE gel and stained with Coomassie stain. (b) A duplicate gel showing LH6 WT and mutant cells after proteinase K treatment and separation on a 12.5% SDS-PAGE gel (part shown in Figure 7d). The gel was stained with Pro-Q™ Emerald 300 glycan stain to visualize capsular polysaccharides (~30-75 kDa) and low molecular weight glycans (<17 kDa).

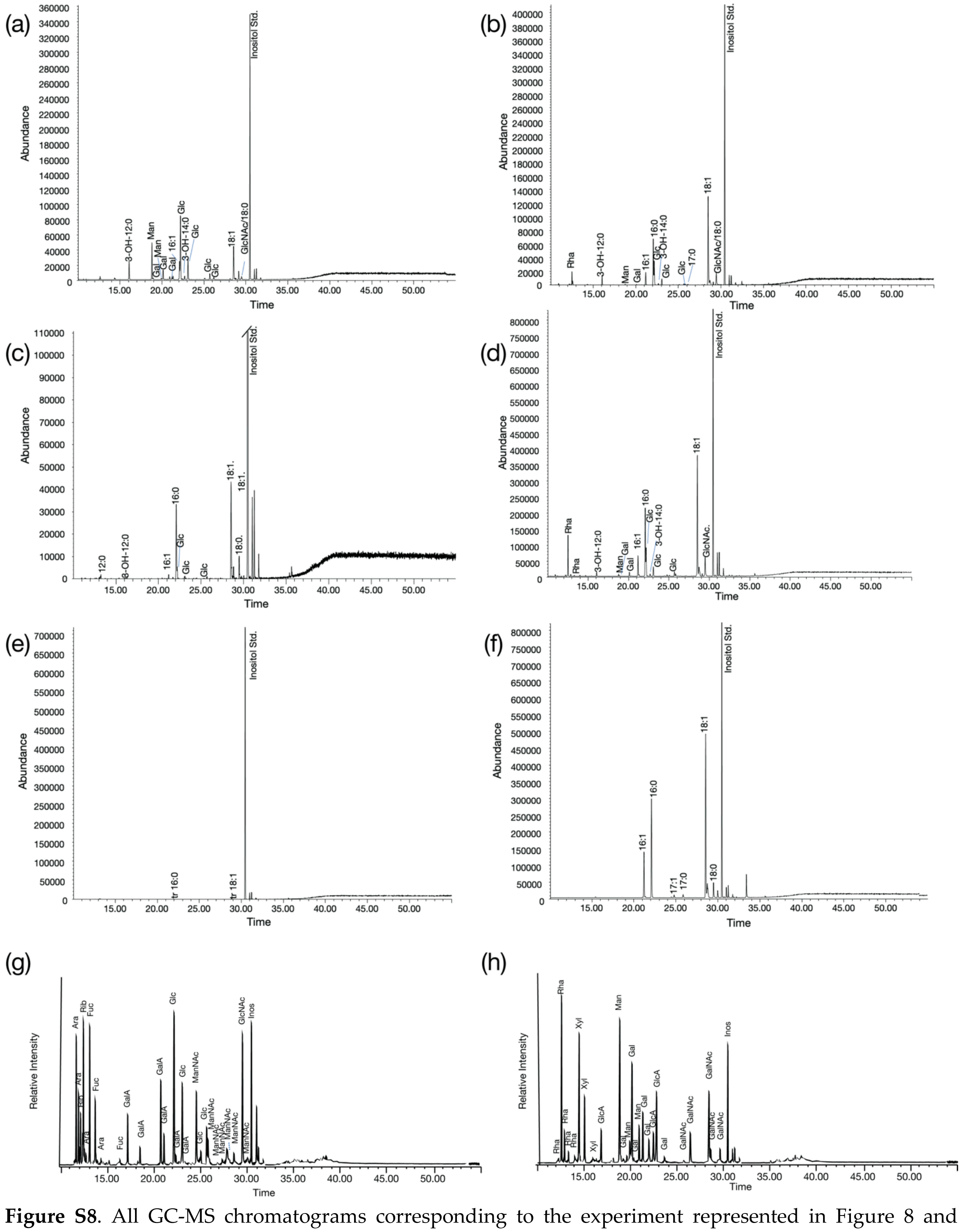

Figure S8. All GC-MS chromatograms corresponding to the experiment represented in Figure 8 and standard monosaccharides. Panel identities are as follows: (a) CAP3 aqueous (b) LH6 aqueous (c) CAP3 interphase (d) LH6 interphase (e) CAP3 organic phase (f) LH6 organic phase. (g, h) Total electron ionization (EI) ion GC-MS chromatograms of monosaccharides converted to trimethylsilyl (TMS) methyl glycosides.

Each monomeric sugar that is converted to TMS-derivatives will form stereoisomers that elute at different retention times. The monosaccharides were grouped into two standard mixtures (g, h) to prevent the co-elution of diagnostic peaks. A relative retention time and the EI mass fragmentation for individual glycosyl residues were used in all analyzed samples to identify the glycosyl residues. Legend: Ara - arabinose, Rib - ribose, Fuc - fucose, GalA – galacturonic acid, Glc - glucose, ManNAc – N-acetylmannosamine, GlcNAc - N-acetylglucosamine, Rha- rhamnose, Xyl- xylose, GlcA – glucuronic acid, Gal – galactose, Man- mannose, GalNAc - N-acetylgalactosamine, Inos - *myo*-inositol.

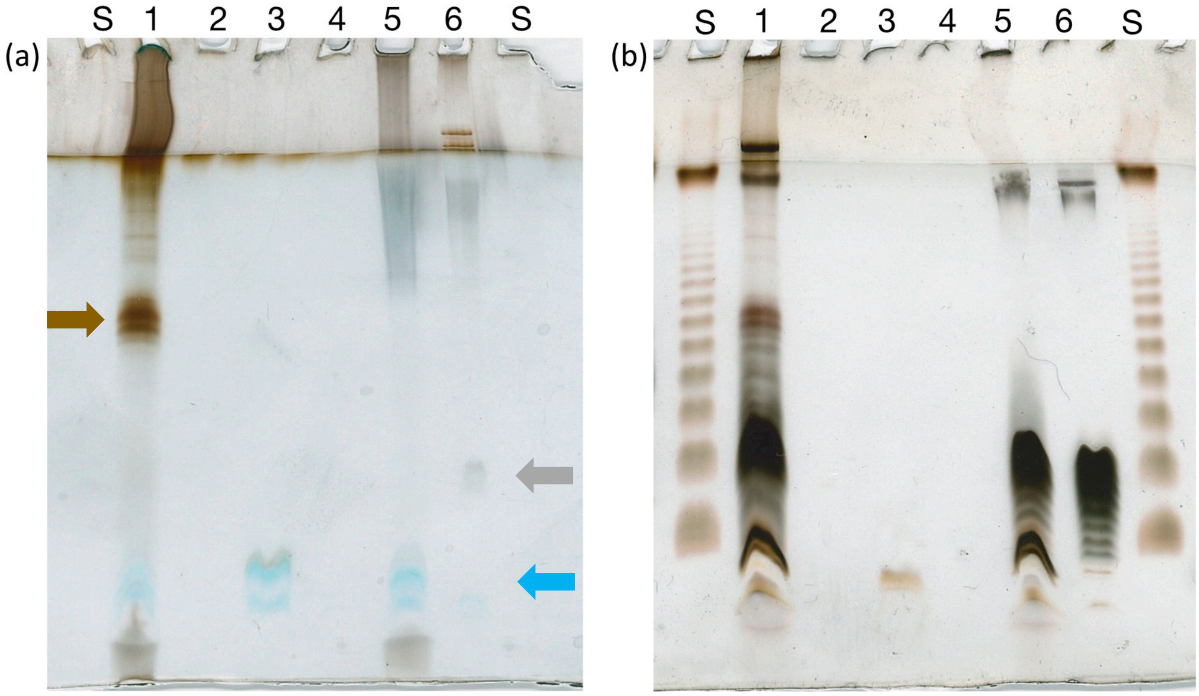

Figure S9. 18% DOC-PAGE analysis of bacterial and phage extracts examined by GC-MS in Figure S8 (and Figure 8). (a) Samples were stained overnight with alcian blue and with silver reagent without periodate oxidation prior to silver staining. (b) Samples were oxidized with periodate followed by silver staining. Lanes: S: *Salmonella enterica* serovar Minnesota S-type LPS; Number labels correspond to the following phases: 1: LH6 interphase, 2: CAP3 interphase, 3: LH6 organic phase, 4: CAP3 organic phase 5: LH6 aqueous, 6: CAP3 aqueous. Brown arrow corresponds to most abundant fragment sizes of LH6 CPS. Blue arrow may represent free lipid A or phospholipids. Grey arrow points to uniquely staining phage component that may correspond to band above LOS in Figure 7c.
